# STIM1 GoF Mutants: Genotype–Phenotype Relationships Across the Stormorken/TAM/YPS Spectrum

**DOI:** 10.3390/cells15100926

**Published:** 2026-05-18

**Authors:** Lara Atzgerstorfer, Magdalena Prantl, Andrea Waldhauser, Isabella Derler, Marc Fahrner

**Affiliations:** Institute of Biophysics, JKU Life Science Center, Johannes Kepler University Linz, 4040 Linz, Austria; lara.atzgerstorfer@jku.at (L.A.); magdalena.prantl@jku.at (M.P.); andrea.waldhauser@outlook.com (A.W.); isabella.derler@jku.at (I.D.)

**Keywords:** SOCE, CRAC channel, STIM1, Stormorken Syndrome, tubular aggregate myopathy, York Platelet Syndrome, Ca^2+^ signaling

## Abstract

Store-operated Calcium (Ca^2+^) entry (SOCE), mediated by stromal interaction molecule 1 (STIM1) and Orai1, is a central pathway controlling intracellular Ca^2+^ homeostasis. Gain-of-function (GoF) mutations in STIM1 cause a spectrum of clinically overlapping disorders historically classified as Stormorken Syndrome (STK), tubular aggregate myopathy (TAM), and York Platelet Syndrome (YPS). However, increasing evidence indicates that these entities could represent a shared disease spectrum rather than distinct conditions. At the molecular level, STIM1 activation is governed by a series of autoinhibitory checkpoints that maintain the protein in a tightly controlled resting state. GoF mutations disrupt these regulatory constraints, leading to dysregulated SOCE activity that is frequently, but not uniformly, associated with constitutive channel activation depending on the specific mutation and cellular context. While many disease-associated variants localize to the EF hand, a highly conserved helix–loop–helix Ca^2+^ binding motif, and the CC1 (coiled-coil 1) domain involved in molecular regulation of STIM1 activation, an increasing number of mutations in the C-terminal region further expands the mechanistic and clinical spectrum. In this review, we summarize current concepts of molecular STIM1 activation and discuss how distinct mutations perturb specific regulatory elements of the protein. By systematically integrating published case reports into a comprehensive overview, including a mutation–phenotype correlation table, we highlight the remarkable variability in and incomplete penetrance of clinical manifestations.

## 1. Introduction

Calcium (Ca^2+^) is the most abundant mineral in the human body, yet less than 1% resides within the intracellular space, with the cytosolic space containing the absolute lowest Ca^2+^ concentration of about 100 nM in contrast to intracellular Ca^2+^ stores like the endoplasmic reticulum (ER) which can reach ~2 mM Ca^2+^ [1]. This steep gradient underlies the role of Ca^2+^ as a universal second messenger, where transient and spatially restricted increases in cytosolic Ca^2+^ encode signals that regulate diverse cellular processes, including gene expression, secretion, muscle contraction, and cell death [1,2]. This overall striking imbalance of the Ca^2+^ distribution within an organism underscores a fundamental biological principle: even subtle disturbances in cytosolic Ca^2+^ concentrations can have profound and often deleterious consequences for cellular and organismal physiology. Tight control of intracellular Ca^2+^ homeostasis is therefore essential. Among different mechanisms, store-operated Ca^2+^ entry (SOCE) represents the dominant and most conserved pathway in non-excitable cells [3]. SOCE describes Ca^2+^ entry from the extracellular space initiated by the reduction in the ER-luminal Ca^2+^ concentration. Upon Ca^2+^ release from the ER—typically initiated by inositol 1,4,5-trisphosphate (IP_3_) following receptor stimulation [4]—the ER-resident Ca^2+^ sensor stromal interaction molecule 1 (STIM1) detects the reduced luminal Ca^2+^ concentration and activates plasma membrane (PM) Ca^2+^ channels. This allows Ca^2+^ influx into the cell, which serves both to replenish ER Ca^2+^ stores and to generate cytosolic Ca^2+^ signals acting as a second messenger [2,4,5].

Dysregulation of SOCE is directly linked to a broad spectrum of human diseases, including immunodeficiency, myopathies, neurodegenerative and cardiovascular disorders, as well as cancer [6,7,8,9,10]. A major component of SOCE is the Ca^2+^ release-activated Ca^2+^ (CRAC) channel. Notably, other channels like TRPC1 and TRPV6 have also been reported to contribute to or modulate SOCE and Ca^2+^ homeostasis [4,11]. The CRAC channel is composed of the ER-resident Ca^2+^ sensor STIM1 and the Ca^2+^-selective pore-forming Orai1 protein in the PM [3,4,12,13]. Mutations in either component can result in gain- or loss-of-function (GoF or LoF) phenotypes with severe pathological outcomes. While CRAC channel LoF mutations may result in a spectrum of immunodeficiency phenotypes ranging from mild immune dysfunction to severe combined immunodeficiency (SCID), GoF mutations in STIM1 give rise to a group of clinically overlapping disorders, including Stormorken Syndrome (STK), tubular aggregate myopathy (TAM), and York Platelet Syndrome (YPS) [6,9,14,15,16,17,18,19].

**Figure 1:**
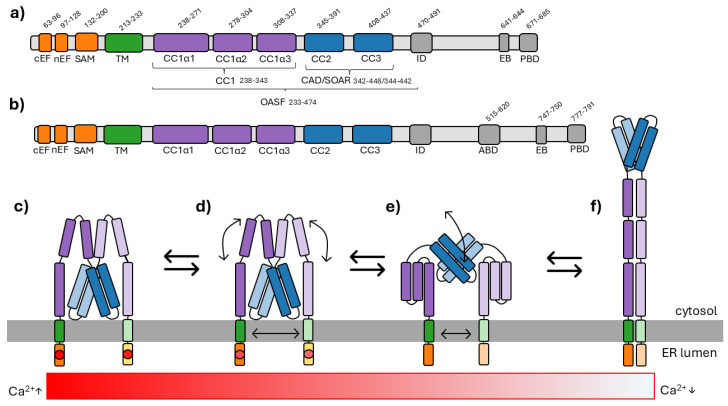
STIM1 domains and activation mechanisms. In panel (**a**) a schematic STIM1 encompassing all the important domains of the protein including their positions is shown. The ER-luminal, N-terminal regions are colored in orange; the TM domain in green; CC1 is shown in purple; CC2 and CC3 are shown in blue; and the domains located at the end of the C-terminus are in gray. In (**b**) the structure of STIM1L is depicted; this STIM1 isoform is extended by an actin-binding domain (ABD), located between the ID and the EB domain. Panels (**c**) to (**f**) illustrate the currently proposed activation mechanism of a STIM1 dimer anchored in the ER membrane, represented by the gray box. In (**c**) the protein assumes its compact Ca^2+^ (indicated via the red circles)-bound resting state. Once the Ca^2+^ concentration in the ER is lowered, the protein shifts through intermediate states. Panel (**d**) shows how CC1α2 and CC1α3 fold down toward CC1α1 in order to form the 3HB. Once the 3HB is formed, CAD reorients from the near-ER membrane toward the PM, as illustrated in (**e**). Finally, the CC1 homomerization leads to a fully activated, extended state after Ca^2+^ depletion from the ER, depicted in (**f**). Structural rearrangements and transitions between the individual conformational states are indicated by arrows. On the bottom, the Ca^2+^ concentration during the activation process is depicted via a gradient representing its depletion from the ER, with high Ca^2+^ concentrations on the left and low Ca^2+^ concentrations on the right. The reaction arrows indicate that this process is Ca^2+^-dependent and reversible, allowing STIM1 to return to its resting state upon refilling of the ER Ca^2+^ stores.

On a molecular level, STIM1 is a dimeric type I single-pass transmembrane (TM) protein predominantly localized to the ER membrane. In addition to the ubiquitously expressed canonical STIM1, alternative splice variants such as STIM1L and STIM1A have been described, which exhibit distinct functional properties and tissue-specific expression patterns. Related homologs, including STIM2 and its isoforms (STIM2.2, STIM2.1 and STIM2.3 [20]), further expand the diversity of STIM-mediated Ca^2+^ signaling and have been comprehensively reviewed by Grabmayr et al. [21]. Among them, the longer STIM1 isoform STIM1L (see Figure 1) has been associated with TAM patients [22]. A schematic representation of STIM1 including all its domains and their positions is depicted in Figure 1. Its luminal N-terminus contains a canonical and non-canonical EF hand (cEF and nEF) followed by a sterile alpha motif (SAM), together forming the Ca^2+^-sensing part of the protein. The TM domain structurally and functionally connects this luminal sensor domain to the cytosolic C-terminus, which comprises three coiled-coil domains (CC1–CC3), an inactivation domain (ID), a microtubule end-binding (EB) domain, and a C-terminal polybasic domain (PBD). The PBD contributes to PM targeting through interactions with the phospholipids phosphatidylinositol 4,5-bisphosphate (PIP_2_) and phosphatidylinositol (3,4,5)-trisphosphate (PIP_3_), thereby facilitating STIM1 accumulation at ER-PM junctions [3,23]. CC1 itself is subdivided into three α-helices (CC1α1–α3), while CC2 and CC3 together mainly form the CRAC activation domain (CAD), also referred to as the STIM1-Orai1-activating region (SOAR), which directly engages with and activates Orai1 [24,25,26]. In addition, CAD/SOAR has been shown to directly interact with phosphoinositides in the PM [27]. Another minimal activation domain is the coiled-coil domain containing region9 (CCb9) comprising residues 339 to 444, similar to CAD/SOAR [28]. An additional important C-terminal region is the Orai1-activating small fragment (OASF), which corresponds to CAD/SOAR extended by CC1, therefore adding a regulatory unit [29].

## 2. STIM1 Activation Mechanism

The conceptual framework for understanding STIM1 activation was established over two decades ago. The earliest works on STIM1 in the context of the CRAC channel focus on the Ca^2+^-sensing ability of the protein, which is linked to its EF-hand domain located in the N-terminal region within the ER lumen [15,16,17]. While the location of the Ca^2+^ binding site was clear early on, the conformational rearrangements linked to binding and unbinding of the ion were resolved a few years later [30,31]. Under resting conditions, the N-terminus of STIM1 assumes a tightly folded and compact state in which the EF hands form a hydrophobic pocket while the SAM domain acts in a stabilizing manner. Following ER Ca^2+^ store depletion and Ca^2+^ dissociation from the cEF hand, the EF-SAM domain destabilizes, unfolds, and oligomerizes, thereby inducing a conformational change that propagates towards the C-terminal region [30,31,32]. Initial studies on the STIM1 C-terminus demonstrated that STIM1 resides in a tightly constrained, autoinhibited resting conformation and undergoes a pronounced conformational extension upon ER Ca^2+^ store depletion [33]. Subsequent work provided critical mechanistic insight by identifying a specific CC1α1–CC3 interaction within the STIM1 cytosolic domain that stabilizes this quiescent state and prevents premature STIM1-Orai1 activation [34,35,36]. In addition, studies revealed activation-dependent CC3–CC3 intermolecular interactions that drive STIM1 oligomerization and cluster formation at ER–PM junctions [29,34]. Complementing these findings, Rathner et al. [37] identified the CC1 domain as a compact three-helix bundle pointing to a structural intermediate state, providing a structural basis for how CC1α1, CC1α2, and CC1α3 cooperate to transmit luminal Ca^2+^ signals throughout the cytosolic domain of STIM1. Additional structural and functional studies refined the molecular understanding of STIM1 regulation. In particular, van Dorp et al. [38] revealed the domain-swapped architecture of the dimeric STIM1 cytosolic region, demonstrating how CC1α1 from one monomer interacts with CC3 of the opposing monomer to form an inhibitory clamp that tethers the CAD/SOAR domain close to the ER membrane. Horvath et al. [39] provided structural support for a swing-out mechanism during STIM1 activation, in which CAD/SOAR undergoes a large-scale rotational reorientation away from the ER membrane toward the PM. Most recently, and grounded in these earlier concepts, Qiu et al. [40] expanded the model by defining four discrete structural “brakes” that collectively stabilize the resting STIM1 dimer and must be sequentially released to permit full activation, culminating in CRAC channel opening. Based on these studies, the now-accepted view of STIM1 activation is a stepwise, energetically controlled process driven by the release of (auto)inhibitory brakes rather than a simple binary switch [34,35,36,37,38,39,40].

In more detail, under store-replete conditions, dimeric STIM1 adopts a compact, autoinhibited conformation and is evenly distributed throughout the ER membrane [24]. Ca^2+^ binding to the cEF hand stabilizes the EF–SAM domain, which together acts as a luminal steric brake [31,41]. On the cytosolic side, the intermolecular CC1α1–CC3 inhibitory clamp restrains CAD/SOAR close to the ER membrane, where additional CAD apex–phospholipid interactions further stabilize the resting state [34,38,40,42]. Finally, the compact CC1α2α3 conformation helps stabilize the dimeric STIM1 tight state, therefore acting as a brake as well [40]. Upon ER Ca^2+^ depletion, Ca^2+^ dissociates from the cEF hand, leading to a destabilization and dimerization of the EF–SAM domains. This conformational signal propagates across the TM domain, driving CC1 rearrangements, i.e., initially forming the intermediate-state CC1α1α2α3 three-helix bundle, forcing CAD dislocation from CC1α1, followed by close CC1α1 homomerization as well as the fully extended CC1 conformation, culminating in reorientation of CAD/SOAR toward the PM [34,37,38,39,40]. Activated STIM1 further oligomerizes via CC3 interactions to stabilize the extended state and efficiently accumulates at ER–PM junctions, where its extended conformation bridges the intermembrane gap and directly engages Orai1 channels to initiate Ca^2+^ influx [24]. To visualize this activation process, Figure 1 schematically shows how STIM1 transitions from its resting state into its extended form following Ca^2+^ depletion from the ER. The minimal functional domain of STIM1 (CAD/SOAR) has long been considered a compact and relatively rigid structural unit, commonly depicted as a dimeric V-shaped structure based on the original description by Yang et al. [43], a view that was further reinforced by structural analyses reported by van Dorp et al. [38]. However, molecular dynamics (MD) simulations challenge this static interpretation by indicating that CC2 is not rigidly locked to CC3 [39]. These findings raise the possibility that CAD/SOAR could undergo dynamic mild conformational rearrangements during STIM1 activation, suggesting that structural plasticity within CAD may contribute to the STIM1 conformational change as well.

This review focuses on GoF mutations in STIM1 associated with STK, TAM and YPS, highlighting how distinct mutations perturb specific autoinhibitory checkpoints within the STIM1 activation pathway. By anchoring disease mechanisms firmly in the established activation framework of wild-type (WT) STIM1, we aim to provide a coherent and mechanistically grounded understanding of how pathological Ca^2+^ signaling emerges when long-evolved regulatory constraints are lost.

## 3. Stormorken Syndrome, Tubular Aggregate Myopathy and York Platelet Syndrome

First published in 1985, Helge Stormorken et al. [44] described a new multifaceted syndrome. They reported a patient and their son presenting with thrombocytopathia, asplenia, muscle contractile defect, migraine-like headache, miosis, dyslexia and ichthyosis. From then on, the autosomal dominant disorder was referred to as Stormorken Syndrome. The association between STIM1 mutations and this disease was first reported in 2014 by several independent groups [45,46,47]. Through the application of targeted as well as whole-exome sequencing on several patients and their unaffected family members, the STIM1 R304W mutant has been identified as the cause of STK. The idea of possible STIM1 involvement arose after Böhm et al. [48] linked TAM to the protein in 2013. Before that, both STK and TAM were already associated with muscle fatigue [49]. Later, in 2015, Markello et al. [50] linked another disease to STIM1 GoF mutations, namely York Platelet Syndrome, which also shares an overlapping phenotype with STK and TAM.

TAM leads to tubular aggregates (TAs) in skeletal muscle fibers. The typical symptoms associated with TAM are muscle weakness and cramps. Patients with TAM can also exhibit myalgia, increased creatine kinase (CK) levels, miosis, thrombocytopenia and thrombocytopathy, hyposplenia, ichthyosis, short stature and dyslexia [6,8,9,14]. YPS patients present with thrombocytopenia, platelet abnormalities and myopathy [50]. The phenotype of STK patients is described as mild bleeding tendency, thrombocytopathia, thrombocytopenia, mild anemia, asplenia, TAs, miosis, headache and ichthyosis, and in some cases, short stature, dyslexia or learning disabilities [6,9,14,22,44,47,51]. Taken together, this shows that these three diseases not only share the same genetic origin in STIM1 mutations but also that their resulting phenotypes show a significant overlap. It is noteworthy that since establishing the link between STIM1 and STK, mutations within Orai1, the pore-forming part of the CRAC channel, and within calsequestrin-1 (CASQ1) have also been linked to the TAM and YPS diseases [8]. CASQ1 is a protein found in the sarcoplasmic reticulum (SR) where it acts as a Ca^2+^ buffer [52]. To date, six Orai1 mutations and five CASQ1 mutations in connection with TAM have been identified [46,53,54,55,56,57,58]. The range of symptoms arising from mutations in these two proteins in particular seems to be primarily centered on TAM with muscle weakness, muscle fatigue and myalgia as the most common phenotypes [46,53,54,55]. Mechanistically, both proteins are key regulators of intracellular Ca^2+^ homeostasis—Orai1 mediating Ca^2+^ influx and CASQ1 controlling Ca^2+^ storage within the SR—and may therefore contribute to tissue-specific phenotypes. However, due to the limited availability of functional and comparative data, such modulatory roles remain speculative. As neither the genetic origin nor the resulting phenotypes of STIM1 GoF mutations associated with STK, TAM, or YPS allow for a clear distinction between these conditions, they will be collectively referred to throughout this review as a single STK/TAM/YPS disease spectrum with variable clinical manifestations.

The symptoms associated with STK/TAM/YPS can vary widely between patients with the same mutation and even more when looking at different mutations causing this disease. Expanding on the work of Morin et al. [9] from 2020, who tried to find a genotype/phenotype correlation, we want to focus on the STK/TAM/YPS mutations identified since and analyze how they fit into the already established picture. In their work, Morin et al. found that phenotypes of mutations within the EF hands of STIM1 varied from clinically asymptomatic to severe muscle weakness starting in childhood and can be accompanied by a single to several other STK/TAM/YPS symptoms. They also showed that mutations confined to the EF hands manifested more mildly, with none of the patients presenting with a full penetration of the STK phenotype. STIM1 R304W, which at that time represented the only C-terminally located STK/TAM/YPS mutation, was found to lead to a more severe phenotype. All patients carrying this specific mutation presented with thrombocytopenia and 88% of them additionally suffered from miosis and 70% also from hyposplenia. A total of 70% of these patients manifested all three of these symptoms [9].

Since this initial in-depth analysis of EF-hand mutations and STIM1 R304W, additional STK/TAM/YPS mutations—particularly several variants located in the C-terminal region—have been identified and further expand the associated phenotypic spectrum. A summary of all patients with STK/TAM/YPS caused by a STIM1 mutation known to date is listed in Table 1. After reviewing all of the case reports, we have identified the most common symptoms associated with STK/TAM/YPS. In Table 1 we summarize how a selected set of symptoms (based on the most frequently found symptoms throughout all case reports) manifest in each patient. For many years, STIM1 R304W represented the only STK/TAM/YPS-associated mutation located in the C-terminal region of STIM1, leading to the assumption that its comparatively severe phenotype, relative to EF-hand mutations, might be linked to its position within the regulatory cytosolic domains of the protein. To better understand how the newly identified C-terminal mutants fit within this framework, their phenotypes are discussed in more detail below. One additional mutation at position 304 (R304Q) was identified as an STK/TAM/YPS mutant, first reported in 2017 by Harris et al. [51]. The symptoms presented by patients carrying the R304Q mutation were most frequently myalgia and stiffness [51]. Although located at the same position as R304W, patients with a Gln substitution at this location showed milder phenotypes than patients with the R304W mutation [14].

In 2016 Okuma et al. [59] examined a patient presenting with slowly progressive muscle weakness and atrophy. They also performed a muscle biopsy and found TAs in the patients’ skeletal muscle leading to a TAM diagnosis. Notably, myalgia, cramps, scoliosis, rigid spine, miosis, thrombocytopenia, asplenia and ichthyosis were ruled out and CK levels were normal. The symptomatic presentation with TAM led them to a genetic analysis which revealed a novel STIM1 frameshift mutation, I484Rfs*21, in the patient as well as his mother, who presented with a walking difficulty. Other healthy family members did not carry the STIM1 mutation.

In 2021 Jiang et al. [60] described a patient presenting with skin purpura in their lower limbs and stroke-like episodes. Following further examination and muscle biopsy, the main symptoms identified in this patient were stroke-like episodes, headache, anemia, thrombocytopenia and bleeding diathesis, mild histological myopathy and short stature. A genetic analysis revealed a novel STIM1 mutation K365N. Their finding of thrombocytopenia as well as anemia and short stature combined with the mutation led them to diagnose the patient with STK, although the patient showed no muscle weakness, hypocalcemia or asplenia as well as having normal CK levels. It is noteworthy that upon treatment of suspected Systemic Lupus Erythematosus with “hormones and sirolimus”, the symptoms and thrombocytopenia resolved [60].

In 2022 another case study with a patient diagnosed with STK/TAM/YPS was published by de la Fuente-Munoz et al. [61], the first describing the L303P mutation within STIM1. The patient was reported as a “clear case of STK/TAM” due to the manifestation of the following symptoms: myopathy, defective dental enamel, numerous dental caries and root canals, brittle nails, no dystrophy, congenital pes cavus of the right foot, congenital hammer toes, arthrosis, generalized myalgia, muscle atrophy with myoclonus and incapacitating fatigue after physical exercise. Notably, the patient’s mother, who suffered from cardiomyopathy, was also reported to carry the L303P mutation but did not exhibit clinical symptoms of STK/TAM/YPS. This observation suggests incomplete penetrance, indicating that additional genetic or environmental factors may modulate the phenotypic outcome of this variant [61].

**Table 1:** This table contains all STIM1 mutations associated with STK/TAM/YPS known to date. The mutations are listed in order of their position within the protein starting from the N-terminus. The table contains information on the case report of each mutation. To gain a clearer understanding of the phenotypes arising from these mutations, the most common STK/TAM/YPS symptoms were matched to the mutants. A green check (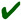) indicates that the symptom was identified in the patient, a red cross (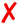) signifies that the symptom was ruled out in the patient, and a black question mark (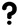) implies that neither occurrence nor absence of the symptom was recorded. Lastly the total number of symptoms identified in a patient is listed.

Patient Number	Mutation	Record	Muscular Weakness	Tubular Aggregates	Increased CK	Thrombocytopenia	Myalgia	Asplenia/ Hyposplenia	Miosis	Hypocalcemia	Bleeding Tendency	Short Stature	Skin Lesions	Number of Symptoms
1	H72Q	[9]	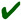	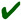	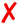	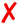	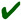	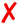	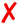	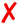	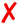	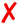	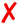	3
2	H72Q	[9]	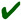	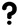	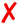	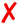	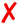	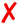	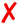	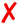	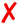	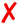	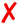	1
3	H72Q	[9]	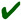	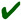	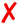	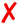	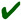	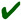	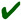	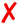	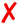	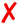	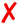	5
4	H72Q	[9]	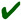	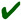	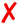	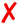	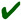	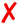	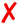	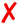	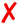	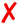	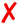	3
5	H72Q	[9]	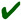	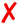	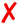	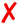	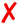	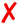	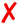	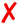	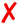	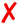	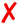	1
6	H72Q	[9]	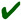	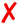	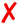	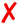	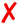	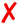	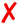	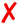	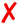	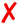	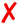	1
7	H72Q	[9]	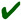	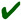	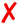	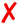	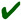	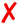	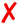	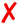	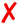	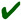	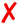	4
8	H72Q	[48]	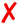	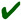	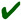	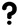	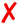	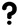	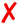	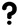	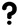	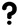	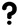	2
9	H72Q	[48]	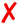	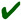	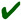	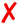	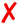	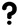	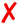	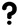	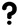	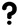	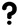	2
10	H72Q	[48]	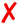	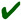	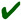	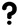	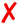	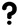	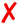	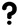	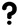	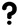	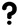	2
11	N80T	[62]	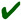	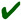	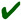	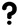	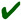	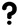	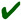	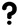	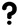	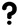	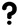	5
12	N80T	[62]	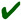	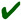	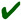	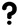	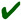	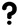	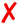	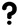	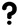	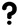	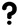	4
13	G81D	[63]	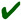	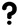	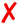	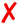	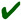	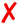	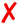	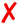	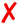	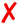	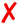	2
14	G81D	[63]	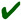	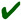	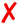	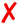	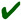	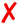	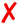	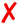	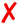	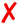	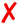	3
15	G81D	[51]	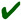	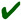	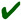	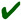	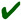	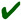	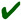	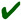	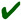	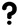	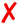	9
16	D84E	[64]	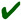	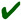	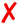	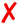	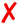	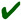	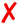	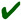	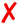	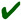	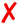	5
17	D84E	[22]	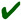	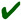	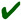	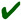	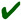	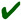	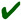	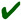	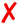	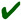	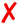	9
18	D84G	[48]	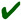	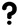	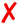	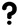	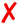	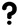	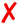	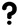	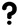	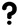	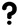	1
19	D84G	[48]	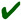	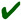	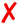	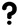	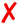	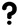	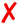	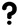	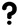	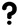	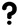	2
20	D84G	[48]	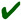	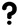	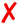	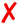	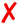	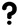	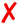	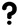	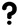	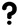	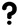	1
21	S88G	[51]	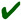	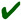	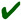	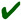	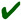	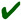	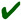	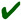	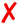	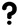	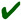	9
22	S88G	[65]	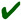	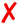	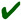	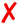	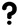	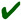	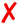	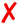	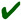	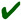	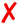	5
23	L92V	[9]	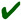	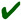	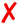	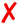	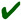	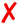	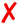	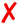	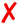	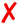	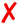	3
24	L96V	[62]	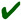	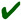	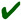	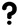	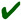	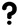	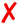	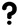	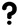	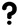	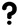	4
25	Y98C	[9]	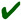	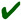	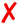	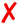	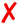	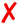	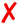	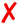	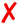	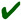	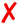	3
26	K104N	[22]	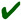	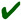	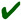	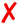	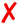	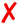	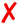	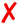	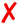	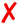	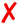	3
27	F108I	[62]	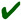	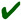	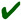	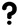	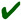	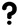	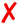	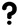	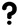	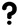	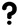	4
28	F108I	[62]	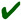	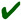	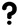	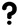	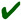	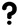	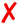	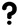	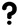	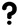	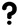	3
29	F108L	[62]	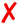	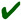	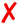	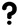	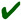	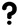	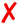	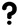	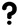	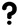	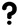	2
30	H109N	[48]	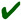	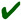	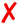	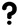	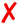	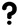	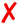	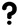	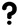	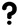	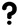	2
31	H109N	[48]	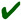	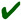	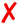	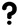	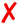	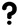	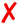	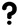	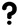	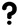	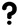	2
32	H109N	[48]	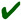	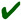	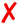	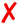	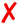	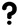	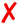	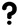	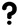	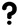	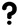	2
33	H109N	[62]	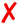	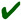	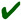	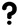	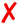	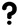	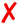	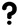	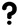	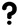	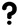	2
34	H109R	[48]	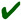	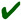	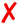	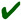	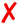	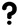	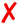	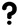	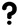	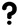	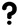	3
35	H109R	[48]	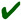	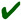	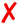	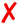	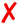	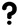	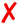	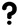	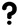	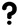	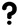	2
36	H109R	[66]	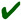	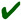	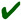	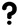	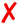	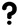	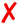	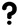	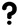	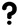	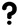	3
37	H109R	[66]	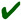	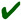	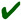	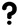	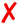	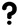	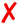	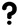	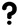	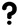	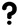	3
38	H109R	[67]	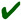	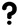	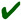	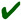	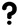	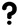	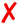	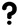	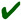	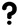	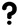	4
39	H109R	[67]	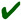	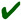	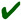	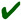	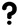	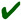	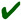	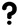	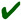	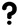	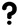	7
40	H109R	[68]	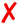	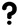	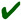	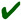	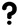	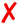	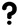	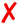	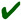	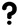	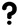	3
41	H109R	[22]	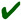	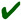	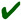	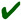	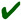	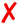	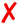	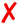	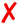	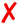	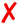	5
42	H109Y	[69]	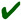	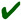	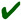	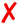	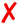	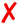	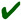	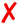	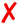	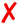	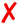	4
43	H109Y	[69]	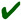	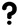	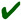	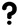	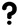	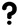	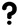	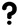	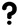	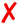	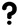	2
44	H109Y	[69]	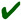	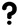	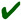	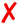	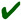	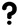	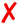	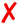	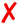	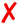	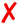	3
45	H109Y	[69]	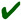	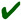	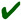	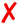	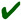	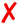	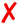	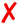	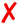	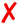	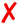	4
46	H109Y	[69]	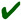	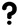	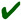	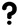	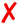	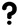	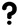	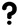	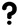	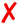	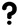	2
47	H109Y	[69]	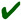	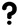	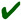	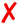	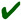	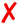	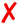	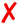	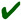	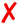	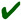	5
48	I115F	[66]	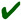	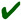	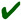	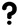	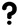	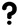	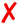	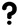	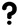	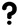	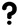	3
49	I115F	[50]	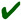	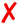	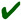	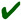	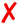	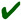	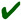	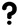	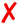	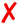	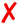	5
50	I115F	[50]	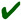	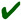	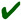	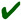	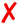	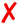	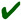	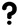	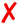	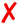	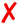	5
51	I115F	[50]	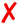	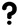	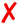	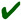	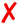	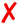	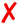	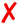	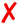	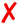	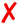	1
52	I115F	[50]	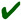	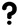	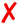	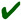	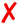	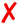	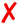	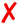	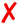	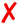	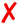	2
53	V138I	[22]	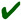	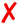	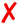	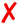	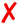	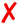	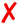	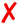	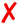	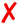	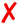	1
54	∆(234–241)	[70]	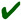	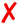	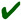	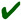	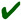	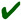	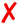	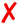	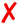	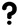	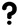	5
55	L303P	[61]	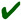	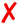	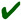	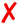	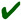	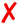	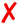	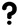	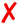	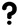	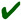	4
56	R304Q	[51]	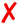	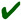	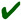	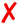	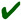	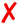	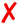	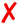	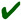	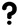	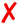	4
57	R304Q	[51]	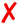	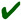	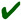	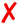	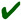	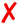	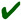	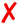	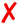	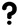	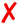	4
58	R304Q	[51]	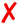	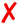	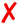	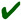	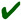	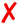	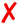	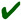	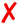	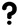	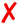	3
59	R304W	[46]	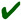	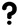	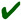	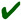	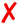	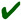	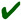	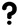	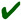	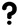	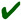	7
60	R304W	[46]	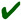	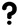	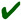	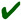	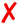	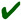	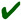	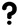	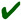	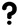	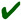	7
61	R304W	[44,71]	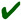	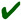	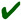	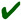	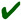	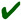	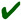	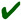	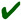	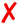	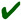	10
62	R304W	[44,71]	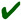	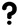	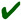	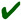	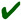	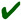	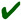	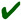	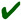	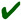	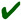	10
63	R304W	[45]	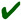	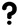	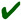	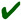	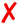	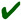	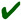	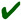	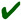	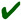	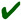	9
64	R304W	[45]	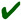	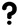	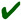	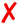	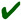	splenectomy	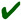	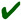	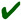	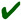	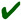	8
65	R304W	[49]	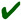	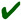	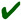	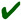	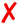	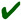	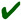	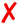	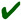	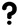	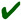	8
66	R304W	[49]	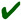	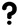	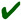	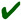	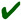	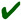	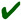	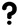	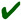	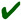	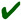	9
67	R304W	[47]	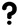	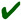	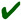	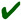	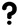	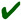	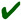	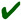	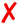	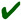	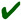	8
68	R304W	[47]	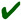	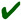	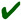	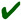	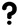	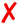	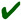	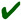	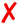	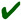	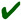	8
69	R304W	[47]	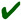	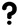	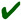	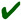	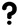	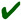	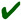	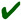	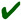	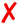	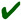	8
70	R304W	[47]	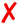	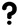	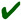	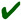	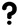	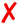	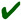	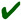	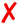	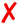	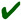	5
71	R304W	[50]	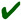	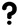	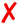	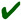	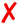	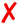	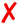	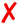	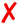	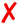	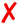	2
72	R304W	[50]	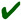	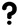	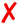	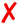	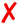	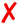	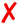	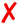	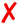	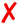	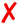	1
73	R304W	[50]	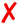	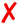	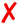	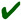	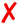	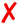	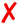	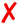	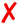	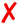	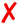	1
74	R304W	[51]	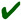	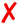	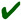	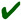	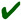	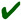	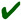	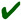	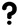	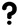	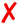	7
75	R304W	[72]	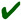	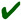	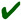	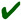	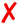	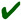	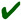	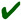	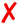	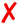	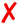	7
76	R304W	[73]	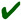	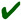	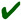	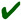	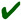	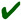	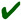	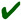	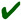	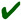	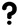	10
77	R304W	[74]	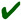	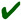	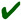	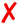	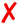	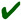	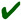	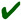	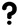	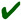	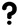	7
78	R304W	[75]	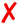	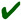	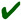	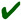	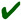	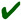	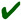	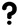	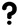	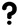	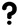	6
79	K365N	[60]	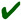	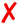	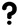	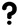	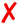	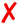	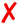	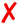	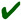	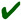	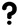	4
80	I484Rfs*21	[59]	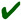	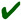	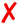	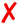	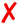	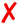	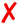	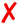	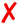	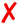	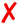	2
81	S630F	[22]	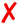	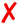	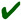	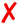	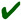	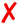	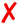	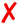	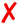	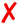	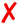	2
82	H632fs*	[22]	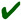	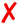	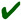	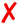	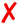	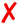	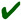	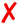	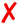	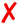	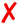	3
83	R749H	[22]	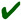	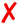	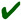	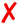	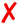	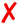	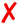	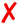	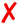	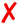	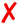	2

Two years later in 2025, the first STIM1 deletion mutation associated with STK/TAM/YPS was reported by Lafabrie et al. [70]. They described a moderate phenotype with symptoms including exercise-induced muscle weakness, elevated CK levels, asplenia and transient thrombocytopenia. The deletion STIM1 ∆(234–241) affected eight amino acids spanning from just after the TM domain to the beginning of the CC1α1 [70].

**Figure 2:**
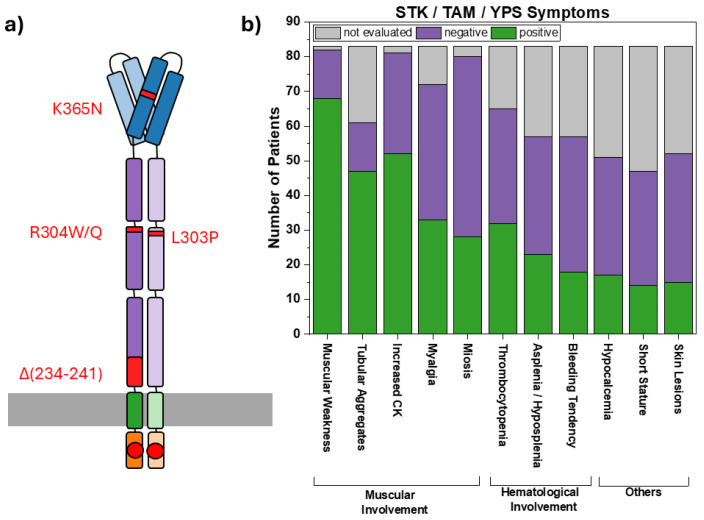
Most common phenotypes across STIM1-mutant STK/TAM/YPS patients. Figure (**a**) depicts a STIM1 dimer under Ca^2+^ replete (red circles) conditions resulting from different GoF mutations recently identified in the C-terminus (positions marked in red) of the protein anchored to the ER membrane (gray box). Panel (**b**) shows a graph listing the most common STK/TAM/YPS symptoms found in the case reports listed in Table 1, containing all STIM1 mutants including the ones located in the EF hands. The graph contains the number of positively (green), negatively (purple) and not evaluated (gray) patients for each of the symptoms.

To further illustrate the resulting phenotype of these STK/TAM/YPS patients, we compiled a bar graph, as shown in Figure 2, depicting how many of the patients listed in Table 1 did/did not manifest each of the most common symptoms associated with this disease spectrum. Additionally, we include the number of unevaluated patients for each symptom. Analysis of the compiled patient data shows that STK/TAM/YPS consistently presents as a disease affecting the muscular system (e.g., weakness, TAs, increased CK levels, myalgia or miosis) and in some cases additionally affects the hematological system (e.g., thrombocytopenia, bleeding diathesis, and a- or hyposplenia) in variable combinations and to variable degrees. This analysis demonstrates that STK/TAM/YPS mutations evoke a very broad phenotypic spectrum. Since not even the most common symptoms develop in all patients, it is very difficult to determine a characteristic set of STK/TAM/YPS symptoms to be used for diagnosis. Furthermore, as Table 1 shows, not even the same mutations lead to a consistent phenotype in different patients. It has been suggested that muscular involvement is more common in EF-hand mutations [9]. However, muscular symptoms have been consistently documented, while hematological symptoms have been evaluated much more frequently in reports of C-terminal mutations and often are not documented in reports of N-terminal mutations (see Table 1).

**Figure 3:**
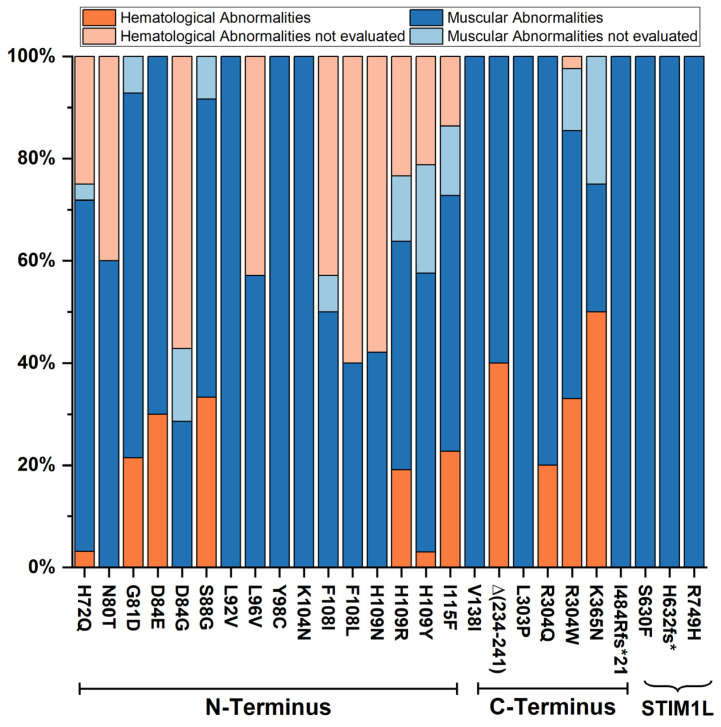
Correlation between mutated positions in STIM1 and muscular/hematological phenotype. This graph shows the mean number of muscular abnormalities (muscular weakness, tubular aggregates, increased CK, myalgia and miosis) and hematological abnormalities (thrombocytopenia, asplenia/hyposplenia and bleeding tendency) for each of the STK/TAM/YPS mutations in dark blue and dark orange normalized to 100%, respectively. Further, the normalized mean number of unevaluated muscular (light blue) and hematologic (light orange) symptoms are depicted for each mutation. This analysis shows that although the evaluated symptoms lead to a correlation of C-terminal mutations with more frequent hematological symptoms, the number of unevaluated symptoms for this phenotype is disproportionately large in the N-terminal region. Therefore, a correlation between muscular or hematological phenotype and N/C-terminal location of the mutation is not possible. Notably, the mutations at the very end of the C-terminus (S630F, H632fs* and R749H) which are part of STIM1L (a STIM1 long isoform) manifest with a purely muscular phenotype.

To further emphasize these observations, Figure 3 shows the mean number of muscular/hematological symptoms found for each of the STK/TAM/YPS STIM1 mutations. Furthermore, it shows how many of the symptoms falling into these two categories were on average not evaluated. The mean values were normalized to 100%. This graph highlights that considering only a subset of the STK/TAM/YPS symptoms during patient evaluation can create a false correlation between the STIM1 position of the mutation and the resulting phenotype. Only considering the evaluated symptoms (depicted in dark blue and dark orange), it would seem that N-terminal mutations are less likely to lead to a hematological phenotype than C-terminal mutations. But the graph illustrates very clearly that for many N-terminal mutations classified with a muscular phenotype, the number of unevaluated hematological symptoms is very high; therefore the phenotype could very likely shift to a more mixed muscular and hematological one if these symptoms were evaluated.

Interestingly, all the mutations found within STIM1L, a STIM1 isoform, show full penetrance of the muscular phenotype. This is probably due to STIM1L being mainly expressed in skeletal muscles [21]. The phenotypes of these STK/TAM/YPS mutations were described with proximal muscle weakness in upper limbs, distal muscle weakness in lower limbs, severe muscle dystrophy with inflammatory infiltrates and elevated CK levels for the patient carrying the STIM1 R749H mutation. STIM1 S630F leads to myalgia and elevated CK levels, while H632fs* is associated with muscle weakness, miosis and elevated CK levels [22].

While the precise mechanisms connecting CRAC channel GoF, specifically in STIM1, to the broad phenotype presented by STK/TAM/YPS patients are not yet fully understood, studies carried out in murine models have provided meaningful insight into the underlying pathophysiology in some cases [76,77,78]. Central to disease mechanisms are the elevated Ca^2+^ levels caused by constitutive SOCE. Grosse et al. [76] demonstrated that the EF-hand mutation STIM1 D84G greatly impacts platelet physiology, leading to preactivated platelets, elevated platelet levels as well as a reduced platelet lifespan. In line with this, Cordero-Sanchez et al. [79] observed a mouse model with the EF-hand mutation STIM1 I115F and found similarities to characteristic TAM symptoms. In a different study, Gamage et al. [77] showed that mice carrying the STIM1 R304W mutation present with reduced growth, skeletal muscle degeneration, and decreased exercise endurance, highlighting the impact of Ca^2+^ dysregulation in muscle function. Contrastingly, Silva-Rojas et al. [78] reported a multisystemic phenotype in mice with the same mutation with a phenotype encompassing muscle weakness, thrombocytopenia, skin and eye anomalies and spleen dysfunction. Interestingly, despite a clear muscle pathology, TAs were not consistently observed in these murine models, pointing to a physiological difference between mice and humans [78,79,80]. As a possible explanation, the authors propose that TA formation in humans may represent an adaptive response to chronic Ca^2+^ overload, possibly trapping excess Ca^2+^ in order to prevent cell damage [78,80].

It should be noted that STK, TAM, and YPS have historically been described as distinct clinical entities, although accumulating evidence suggests that they are likely to represent manifestations within a shared disease spectrum caused by STIM1 GoF mutations. As a consequence, many case reports focus primarily on the diagnostic features of one of these conditions and therefore do not systematically assess the full range of symptoms associated with the STK/TAM/YPS spectrum. For further research regarding STK/TAM/YPS, an extensive standardized clinical and laboratory examination would be highly beneficial for adequate classification of new cases as well as a more accurate understanding of the phenotypical spectrum and pathophysiological mechanisms. A detailed record of patient history and thorough examination regarding the muscular and hematological abnormalities that have been linked to STK/TAM/YPS should be conducted in every case, and it would be helpful if other recurring symptoms of STIM1 GoF such as dyslexia and learning difficulties, short stature and skin lesions were also regularly assessed. Absence of certain symptoms should be documented as well.

## 4. Mechanistic Basis of the STIM1 R304W Mutation

Following the identification of the STIM1 R304W mutation as a cause of STK, several studies have sought to elucidate how this substitution at position 304 alters STIM1 function, leading to constitutive activation of the CRAC channel. Collectively, these studies demonstrate that R304W drives STIM1 into an activated conformation independent of ER Ca^2+^ store depletion, resulting in persistent interaction with ORAI1 and sustained Ca^2+^ influx through CRAC channels [45,46,47,81].

**Figure 4:**
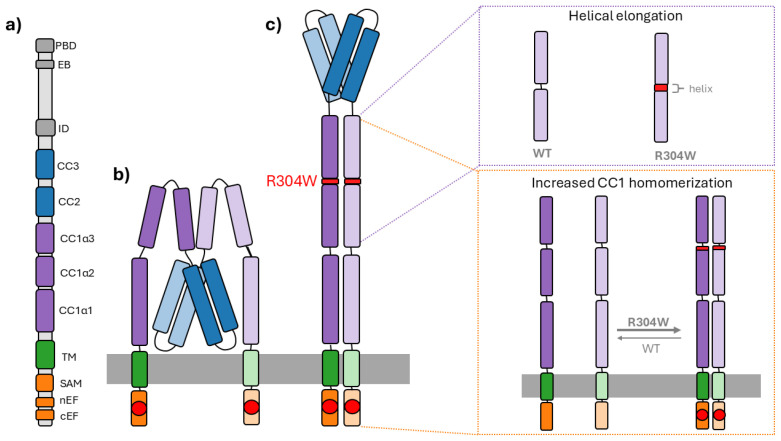
Impact of the STIM1 R304W mutation on the protein structure. In this figure, the STIM1 domain architecture is shown in (**a**) Orange regions represent the ER luminal domains, including the Ca^2+^ sensor domains, while the TM domain is shown in green. The CC1 region is depicted in purple and subdivided into CC1α1–α3. CC2 and CC3 are shown in blue and functionally correspond to CAD/SOAR. The C-terminal regions are illustrated in gray. In (**b**) a STIM1 dimer in its resting state is depicted, while the protein in (**c**) represents the activated conformation due to the STIM1 R304W mutation (at the end of CC1α2), shown in red. The red circles on the N-terminus indicate the Ca^2+^-bound state in ER-replete conditions. The zoom-ins on the right side of the figure show the R304W dependent helical elongation of CC1α2 on top and the increased CC1(α1) homomerization on the bottom, which together lead to the constitutively extended conformation of the dimer (indicated by the arrow as the predominant structural state).

The R304W substitution is located in the cytosolic portion of STIM1 at the very end of CC1α2, i.e., where the loop region connecting the CC1α2 and CC1α3 helices starts [24,37]. For a considerable time, R304W was thought to represent the only C-terminal STIM1 mutation associated with STK/TAM/YPS, whereas all other known disease-causing variants were confined to the luminal EF-hand domains, interfering with Calcium sensing [6]. Electrophysiological analyses established that STIM1 R304W induces constitutive CRAC channel currents, comparable to maximal WT CRAC channel currents, thereby classifying it as a robust GoF mutation [45,46,47,81]. Furthermore, and to dissect the underlying activation mechanism of STIM1 R304W, fluorescence resonance energy transfer (FRET)-based approaches were employed to monitor and analyze conformational changes in STIM1 [81]. These experiments, conducted using both full-length STIM1 as well as STIM1 fragments, revealed that R304W robustly destabilizes the resting, inactive conformation of STIM1 through two principal mechanisms: First, the mutation enhances CC1 homomerization, mostly via CC1α1, promoting intermolecular interactions characteristic of the activated, extended state of STIM1. Second, R304W induces a pronounced α-helical elongation of CC1α2, thereby reducing the length of the linker region connecting CC1α2 and CC1α3 [37,81]. A schematic representation of how this mutation affects the structure of STIM1 is depicted in Figure 4. Thus, the substitution of the hydrophilic and positively charged arginine with the bulky and highly hydrophobic tryptophan residue drastically reduces the flexibility of this loop region, thereby inhibiting proper folding of the adjacent α-helices due to diminished degrees of freedom. In the structural framework proposed by Qiu et al. [40] (based on pioneering studies by Van Dorp et al. [38] and Rathner et al. [37]), the CC1α2–CC1α3 region constitutes one of four discrete autoinhibitory checkpoints that collectively govern the structure–function relationship of STIM1. The R304W mutation disrupts this brake, favoring and supporting the extension of the cytosolic domain of STIM1. Together, increased CC1 homomerization and CC1α2 helix elongation cooperatively destabilize the autoinhibited state of STIM1 and permanently shift the protein toward its extended, active conformation. As a consequence, the CC1α1–CC3 inhibitory clamp is not realized, allowing the CAD/SOAR apex to project toward the PM and constitutively engage ORAI1.

Additional work by Gamage et al. [42] supported by MD simulations has further refined this STIM1 R304W activation model by demonstrating that the GoF characteristic of STIM1 R304W additionally depends on an aberrant molecular interaction with a residue located within the CC1α1 helix. Structural and functional analyses indicate that the bulky and hydrophobic tryptophan at position 304 enables a short-lived interaction with L248, which transiently couples the CC1α2/α3 region to CC1α1, preventing formation of the CC1α1–CC3 inhibitory clamp that normally maintains STIM1 in its resting state [42]. Collectively, these data support a model in which the STIM1 R304W mutation actively stabilizes a pathological, store-independent, active conformation of the protein by at least three effects induced by tryptophan at amino acid position 304 [42,81], culminating in strong and persistent GoF of the CRAC channel, thereby providing a direct molecular explanation for the multisystemic manifestations observed in patients with STK in the presence of STIM1 R304W.

In this context, it is noteworthy that an alternative substitution at the same position, R304Q, has also been reported to promote store-independent STIM1 activation, albeit with clearly attenuated efficacy compared to R304W. Functional analyses demonstrate that STIM1 R304Q enhances STIM1 activity and SOCE, consistent with a partial destabilization of the STIM1 tight state, while lacking tryptophan (R304W)-specific effects and interactions [8,82]. Accordingly, R304Q has been associated with milder clinical manifestations compared to R304W, reviewed in [7,60]. Together, these observations identify residue 304 as a critical modulatory hotspot within CC1, where the properties of the substituting amino acid fine-tune the extent of STIM1 gain-of-function and thereby shape disease severity within the STIM1-mutant-based pathologies.

## 5. Mechanistic Basis of C-Terminal STIM1 STK/TAM/YPS Mutations

In many case reports describing newly identified STIM1 mutations in patients presenting symptoms within the STK/TAM/YPS spectrum, parallels are often drawn to the well-characterized effects of the STIM1 R304W mutation. From the analysis of the molecular mechanism of STIM1 R304W, we have already established that this mutation integrates two very distinct structural and functional properties that lead to the conformational changes in STIM1 resulting in GoF. Notably, STIM1 R304W promotes a helical extension within the loop region connecting CC1α2 and CC1α3, a structural rearrangement that is confined to a very specific segment of the protein. It is therefore not necessarily expected that mutations located in other regions of the STIM1 C-terminal part will induce constitutive activation through the same molecular mechanism.

In their 2021 case report, Jiang et al. [60] described an STK/TAM/YPS patient carrying the STIM1 mutation K365N. Based on the patient phenotype, they proposed this variant to represent a GoF mutation of STIM1. However, as the study did not include direct functional or mechanistic analysis, the precise molecular mechanism underlying the proposed GoF effect remains unresolved. Based on its location in the middle of CC2 within the CAD/SOAR domain, the authors suggest that K365N could destabilize the inhibitory clamp of STIM1 in a way that leads to its release and activation of STIM1 [60]. In this context, a study by Shrestha et al. [83] provides useful insight into the role of position K365. Using a FRET-based two-component system, their alanine scan identified several residues within CC2 as contributors to the stabilization of the resting STIM1 conformation. Although alanine substitution at K365 did not abolish clamp formation, it reduced FRET compared to WT, indicating that this position participates in the maintenance of the resting state [83]. While these findings do not define the mechanism of K365N directly, they are consistent with the hypothesis that perturbation of this site may weaken the inhibitory clamp, which functions as a key autoinhibitory “brake” within the STIM1 activation pathway [40], thereby facilitating CAD release and causing constitutive CRAC channel activity.

De la Fuente-Munoz et al. [61] identified STIM1 L303P in a patient with STK/TAM/YPS and used structural modeling to show that the side chain of L303 is oriented toward the interface between the two α-helices of the CC1α3 region, based on the study by Cui et al. [84]. To assess the functional consequences of the variant, Ca^2+^ signaling was analyzed in peripheral blood mononuclear cells from the patient and three healthy donors. However, the data did not convincingly demonstrate a GoF phenotype comparable to that characteristic of the STIM1 R304W mutation, since a significant cytosolic Ca^2+^ increase was only observed after store depletion [46,81]. Given the limited sample size, additional studies, including experiments in transfected heterologous cell systems, would be needed to verify the molecular mechanism induced by L303P and provide a more comprehensive view of the variant’s functional impact. Given the L303P mutant’s immediate proximity to the well-characterized R304W mutant, it is tempting to speculate about comparable GoF mechanisms. However, the physicochemical properties of the substituted amino acids argue against an analogous effect. While R304W has been proven to extend the CC1α2 helix and rigidify the adjacent loop region [37,81], proline is classically viewed as a helix-breaking residue and would therefore be expected to exert a distinct local effect [85]. In line with this consideration, secondary structure prediction for STIM1 L303P does not reveal major deviations from WT STIM1, in contrast to STIM1 R304W [81,86]. Notably, electrophysiological recordings by Fahrner et al. [81] demonstrate that STIM1 L303W, when co-expressed with Orai1 in HEK293 cells, gives rise to constitutive Ca^2+^ inward currents that are initially somewhat smaller than those observed for R304W and remain partially store-dependent, yet ultimately reach slightly higher maximal amplitudes. Considered alongside the findings of de la Fuente-Munoz et al., these results suggest that increased rigidity within the CC1α2-CC1α3 region, as introduced by tryptophan (R304W), may favor a more store-independent GoF, whereas the distinct steric effects introduced by proline (L303P) may instead result in a GoF phenotype that can still retain store dependence [61,81]. Taken together, these observations highlight the need for further experimental analysis to resolve the molecular mechanism of the L303P mutation, as its effects cannot be readily inferred from the well-characterized R304W mechanism.

Unlike the STK/TAM/YPS-associated STIM1 variants discussed so far, which are caused by point mutations, STIM1 ∆(234–241) represents an in-frame deletion variant. In their study, Lafabrie et al. [70] investigated its functional consequences in murine and human cell lines and found that STIM1 ∆(234–241) forms clusters and already recruits Orai1 under resting cell conditions. Moreover, when co-expressed with WT STIM1, the deletion mutant results in overlapping mutant and WT clusters, indicating that STIM1 ∆(234–241) can associate with and recruit WT STIM1 into mixed oligomers. Furthermore, Lafabrie et al. used NFAT reporter assays and Ca^2+^ imaging to compare STIM1 ∆(234–241) with the STIM1 R304W mutant. In both readouts, the deletion variant showed a phenotype comparable to that of R304W, leading the authors to suggest that these variants may share related molecular pathophysiological mechanisms. Given that the deletion mutation affects the beginning of CC1α1, a region critical for maintenance of the inhibitory clamp, they propose that STIM1 ∆(234–241) may shift the protein toward the active state by destabilizing or releasing this autoinhibitory interaction [70]. However, recent structural models have highlighted the importance of interactions between the CAD/SOAR apex and the ER membrane as one regulatory constraint during STIM1 activation [39,40]. In these models, CAD/SOAR–ER membrane interactions contribute to stabilizing the resting STIM1 conformation. In this context, it is possible that the ∆(234–241) mutation, located at the beginning of CC1α1, may alter the spatial positioning of CAD/SOAR relative to the ER membrane, thereby relieving this regulatory constraint and promoting a constitutively active conformation. Although this scenario remains speculative, it represents a mechanistically plausible explanation for the observed GoF phenotype and could be addressed in future structural or functional studies.

In a patient presenting with TAM, the frameshift mutation I484Rfs*21 was identified in the distal C-terminal region of STIM1, introducing a premature stop codon. In stark contrast to variants located within CC1 or the CAD/SOAR domain, functional characterization by Okuma et al. [59] indicates that this variant does not promote constitutive activation at all, but instead results in aberrant perinuclear aggregation and markedly reduced Ca^2+^ influx, consistent with impaired SOCE. Mechanistically, these findings suggest that truncation of the C-terminus interferes with proper intracellular localization and/or stability of STIM1, thereby preventing efficient coupling to Orai1. This behavior clearly differs from classical GoF mutations such as R304W, which enhance STIM1 activation through defined conformational rearrangements within CC1. The I484Rfs*21 variant therefore represents a mechanistically distinct case in which disruption of C-terminal integrity leads to defective STIM1 function rather than constitutive activation. How this apparent LoF-like phenotype gives rise to TAs, a hallmark of TAM typically associated with STIM1 GoF, remains unresolved and points toward additional pathogenic mechanisms.

Interestingly, additional variants (S630F, H632fs*, and R749H) located in the distal C-terminal region have been reported by Ticci et al. [22] to cause STK/TAM/YPS. In contrast to previously described disease-associated mutations, these variants occur within the long STIM1 isoform (STIM1L), thereby highlighting STIM1L as a disease-relevant isoform. First, HEK cells expressing STIM1 S630F and STIM1 R749H exhibited elevated constitutive basal Ca^2+^ levels compared to WT controls, consistent with enhanced basal CRAC channel activity. In addition, Ca^2+^ imaging measurements showed significantly increased Ca^2+^ entry following store depletion for both variants. No functional characterization was provided for the STIM1 H632fs* mutation. STIM1L is predominantly expressed in skeletal muscle, where its extended C-terminus contains an actin-binding domain that enables pre-assembled STIM1–Orai1 complexes and thereby supports the very rapid activation of SOCE required for repetitive Ca^2+^ signaling during muscle activity [21]. Consequently, perturbations within this STIM1L-specific C-terminal region may directly interfere with this specialized Ca^2+^ signaling machinery and promote unbalanced CRAC channel activity, providing a plausible mechanistic link to the myopathic phenotype observed in STK/TAM/YPS. Notably, STIM1L is also expressed in the brain, raising the possibility that mutations affecting this isoform may additionally impact neuronal Ca^2+^ signaling and thereby contribute to neurological features occasionally observed in the clinical spectrum of these disorders [21].

## 6. Conclusions

Over the past several years, substantial progress has been made in understanding the molecular mechanisms underlying STIM1 activation and its role in SOCE. GoF mutations in STIM1 have revealed how disruption of this tightly regulated process can result in altered CRAC channel activity and dysregulated Ca^2+^ homeostasis, thereby giving rise to a clinically interconnected disease spectrum encompassing Stormorken Syndrome, tubular aggregate myopathy, and York Platelet Syndrome. Rather than representing distinct entities, these conditions are best understood as manifestations of a shared pathophysiological continuum.

Despite these advances, key questions remain unresolved. The precise mechanisms by which different mutations alter STIM1 regulation and Ca^2+^ signaling are not fully understood, and genotype–phenotype relationships remain difficult to define. Importantly, GoF mutations in STIM1 do not uniformly translate into increased SOCE, and disturbances in Ca^2+^ homeostasis can manifest differently depending on the specific mutation, cellular context, and downstream signaling pathways affected. In addition to altered Ca^2+^ influx, changes in ER/SR Ca^2+^ store content and Ca^2+^-dependent signaling processes may further contribute to tissue-specific functional impairment. Identical mutations can give rise to highly variable clinical manifestations, and even the most frequently reported symptoms are not consistently observed. This phenotypic heterogeneity and incomplete penetrance complicate both disease classification and diagnosis.

Several limitations of the current knowledge base must be considered. Most available data are derived from single-patient case reports or small cohorts, limiting statistical power and systematic analyses. Clinical characterization is often incomplete or inconsistent, with many studies focusing on selected features rather than assessing the full disease spectrum. In addition, mechanistic insight remains restricted, as detailed functional analyses are only available for a limited number of variants, leaving many genotype–phenotype relationships unresolved.

Addressing these challenges will require more standardized and integrative approaches. Comprehensive clinical phenotyping combined with systematic functional characterization of STIM1 variants will be essential to refine genotype–phenotype correlations and to better understand the molecular basis of tissue-specific disease manifestations. Expanding analyses to include interacting components of the Ca^2+^ signaling machinery may further help to explain the variability in clinical outcomes.

In summary, while significant progress has been made, a complete understanding of how specific STIM1 mutations translate into clinical phenotypes will require coordinated efforts integrating clinical, genetic, and mechanistic molecular data.

## Data Availability

No new data were created or analyzed in this study. Data sharing is not applicable to this article.

## Abbreviations

ABD	actine-binding domain
Ca^2+^	Calcium
CAD	CRAC activation domain
CASQ1	calsequestrin-1
CC	coiled-coil
CK	creatine kinase
CRAC	Calcium release-activated Calcium
EB	end-binding
cEF	canonical EF
nEF	non-canonical EF
ER	endoplasmic reticulum
FRET	Förster resonance energy transfer
GoF	gain-of-function
ID	inactivation domain
IP3	inositol 1,4,5-trisphosphate
LoF	loss-of-function
MD	molecular dynamics
OASF	Orai1-activating small fragment
PBD	polybasic domain
PM	plasma membrane
PIP_2_	phosphatidylinositol 4,5-bisphosphat
PIP_3_	phosphatidylinositol (3,4,5)-trisphosphate
SAM	sterile alpha motif
SCID	severe combined immunodeficiency
SOAR	STIM1-Orai1-activating region
SOCE	store-operated Calcium entry
SR	sarcoplasmic reticulum
STIM1	stromal interaction molecule 1
STK	Stormorken Syndrome
TA	tubular aggregate
TAM	tubular aggregate myopathy
TM	transmembrane
TRPC	transient receptor potential canonical
TRPV	transient receptor potential channel of the vanilloid subtype
WT	wild-type
YPS	York Platelet Syndrome
